# Severe Acetaminophen Toxicity From the Use of Oxycodone-Acetaminophen With Normal Liver Function Tests and a Completely Asymptomatic Course of Hospitalization

**DOI:** 10.14309/crj.0000000000001126

**Published:** 2023-08-17

**Authors:** Muhammad Adnan Haider, Yousra S. Gheit, Talwinder Nagi, Charles Vallejo, Zoilo K. Suarez, Oscar L. Hernandez, Polina Gaisinskaya, Nathan Markwart

**Affiliations:** 1 Florida Atlantic University Hospital, Schmidt College of Medicine, Boca Raton, FL

**Keywords:** acetaminophen, N-acetylcysteine, fomepizole, APAP, Rumack-Matthew nomogram, oxycodone, hepatotoxicity

## Abstract

Acetaminophen (N-acetyl-p-aminophenol, APAP), after being metabolized to toxic N-acetyl-p-benzoquinone imine, can cause irreversible hepatic necrosis. The mainstay of treatment includes N-acetylcysteine and fomepizole or liver transplant in patients who further deteriorate. Currently, many overdoses unintentionally occur in the setting of ingesting combined products that contain APAP. We report a rare case of a 60-year-old woman who presented with altered mental status and APAP toxicity in the setting of oxycodone-APAP overdose. She had a toxic serum APAP level on arrival. During hospitalization, her APAP level remained at the toxic level on the Rumack-Matthew nomogram. However, her liver function tests remained within normal limits, and she remained completely asymptomatic. To our best knowledge, this is the second case report with asymptomatic APAP toxicity and normal liver function tests. We will explore the effect of concomitant oxycodone ingestion on possibly delaying APAP absorption and thus resulting in a more favorable prognosis without hepatotoxicity.

## INTRODUCTION

Oxycodone is a mu-type opioid receptor agonist that is often prescribed alone or in combination with acetaminophen (APAP). Adverse effects of oxycodone include sedation, respiratory depression, constipation, addiction, and adrenal insufficiency. Liver injury may also occur in the setting of excess APAP ingestion. In 2014, the Food and Drug Administration advised against opioid combination medications in which the dose of APAP is greater than 325 mg per tablet, because of hepatotoxicity.^1^

APAP toxicity is one of the most common causes of liver failure in both the United States and the United Kingdom.^2^ Approximately half of these cases are unintentional overdoses, namely in the context of unknowingly consuming medications that are combined with APAP. Typically, APAP is rapidly absorbed from the gastrointestinal tract and reaches therapeutic levels within 30 minutes to 2 hours. Overdose levels peak at 4 hours unless APAP is coingested with an agent that slows gastric motility, such as an opiate.^3^

Primarily, if the overdose time is known, gastrointestinal decontamination measures in an alert patient may be used. Probable hepatotoxicity can be best predicted by a toxic APAP level in acute overdose or abnormal liver transaminases in a delayed presentation of acute overdose.^4^ N-acetylcysteine (NAC) is used as a prophylactic or treatment of APAP toxicity.^5^ It is indicated when serum levels fall in the toxic range as illustrated by the Rumack-Matthew nomogram. Fomepizole is also used and is believed to inhibit the formation of toxic APAP metabolites and may prevent mitochondrial toxicity. If the patient continues to deteriorate, particularly exhibiting renal failure, metabolic acidosis, encephalopathy, or coagulopathy, then transplant should be considered.^6^

Although there are several studies examining the relationship between APAP and hepatotoxicity, there are few studies focusing on APAP overdose in the context of combination product such as oxycodone-APAP, used for moderate-to-severe acute or chronic pain. Some studies discussed below suggest that coingested oxycodone may delay APAP absorption and, therefore, may help explain the resulting normal liver function tests (LFTs) and favorable prognosis, despite toxic serum levels of APAP, as discussed in this case report.^7^

## CASE REPORT

A 60-year-old woman with a medical history of anxiety, depression, and chronic drug use presented to the emergency department from an assisted living facility after she was found unresponsive by staff. APAP pills were found nearby. The staff was unaware of the patient's time since she was unresponsive and whether she had ingested APAP. En route to the emergency department, the patient received 0.4 mg of Narcan intramuscularly, by emergency medical services. On arrival at the facility, she continued to exhibit altered mental status and received an additional 1 mg of Narcan. Subsequently, the patient became more alert and oriented.

Initially, the patient's serum APAP level was 271 μg/mL, and probable time from ingestion was approximately 4 hours, on the basis of research from staff. Her physical examination was unremarkable at bedside. She was hemodynamically stable, and arterial blood gas reported pH 7.37, CO_2_ 31 mm Hg, and O_2_ 108 mm Hg. In addition, blood urea nitrogen was 21 mg/dL, creatinine 1.14 mg/dL, and creatine phosphokinase 623 U/L. Ammonia levels were within normal limits. Urine drug screen was positive for opiates, benzodiazepines, and tetrahydrocannabinol.

Poison control was contacted, and she received NAC and fomepizole per their recommendations. Subsequently, the patient was admitted and continued to receive care in the intensive care unit. The repeat APAP levels were 306 μg/mL, 275.4 μg/mL, 92.3 μg/mL, 18.9 μg/mL, and <10 μg/mL at 12, 24, 36, 48, and 60 hours, respectively. This is presented in Table 1 with LFTs. The patient remained within toxicity levels on the Rumack-Matthew curve. She continued to receive additional doses of NAC until reaching the following goals:

 1. APAP level becomes undetectable.
2. LFTs rise to the peak and then drop to <50% of peak LFTs.

**Table 1.** Time (hours) since the patient's overdose of APAP and the corresponding APAP level, PT, INR, and ALT and AST

Time (hr)	APAP level (μg/mL)	PT (sec)	INR	ALT (U/L)	AST (U/L)
0 (at presentation)	271	16.6	1.4	14	40
12	306	17	1.4	19	42
24	275.4	21.2	1.9	17	31
36	92.3	23.8	2.1	15	27
48	18.9	19	1.6	14	30
60	<10	17.2	1.4	15	29

ALT, alanine aminotransferase; APAP, acetaminophen; AST, aspartate aminotransferase; INR, international normalized ratio; PT, prothrombin time.

**Figure 1.**
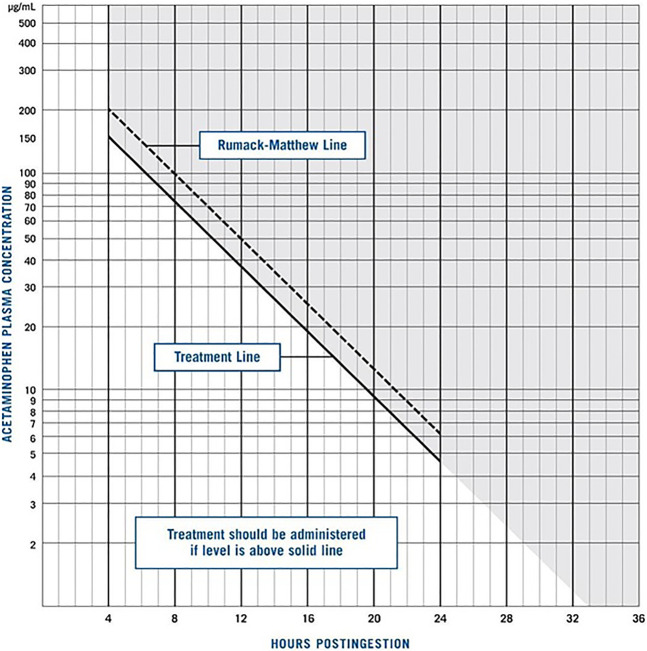
Rumack-Matthew nomogram. The patient's acetaminophen level is plotted at 271, 306, 275.4, and 92.3 μg/mL at hours 0, 12, 24, and 36, respectively, since ingestion.

The patient's LFTs remained normal throughout her hospitalization, and she remained asymptomatic. However, at 24 hours, her APAP level was 275.4 μg/mL, classified as an extremely toxic level. Her APAP level at different time lines is drawn on the Rumack-Matthew nomogram, as shown in Figure 1. As per the literature, the maximum toxicity happens between days 2 and 3. Therefore, the Liver Transplant Center was contacted preemptively, in case the patient experiences liver failure. Fortunately, the patient remained asymptomatic, and her LFTs remained normal after 3 days. Ultimately, the patient was transferred from the intensive care unit to regular medicine floors, and her hospital course remained uneventful. She was discharged to rehab on day 5.

## DISCUSSION

Although APAP-associated hepatotoxicity is reported through various studies, concomitant opioid ingestion is examined only through a few case reports. It is believed that opioids cause a delay in gastric absorption of APAP and, therefore, alter the pharmacokinetics and time of APAP to reach peak concentration levels in the serum. As a result, there may be 2 concentration peaks. Administration of NAC until APAP levels are undetectable may lead to consistently normal LFTs, prevention of hepatotoxicity, and a favorable prognosis of APAP overdose. In our case, the initial APAP level was 271 μg/mL and increased to 306 μg/mL within 12 hours. As aforementioned, this is likely from the delayed absorption of APAP when coingested with opioids, namely oxycodone. Notably, we continued treating the patient with NAC, despite being asymptomatic and exhibiting normal LFTs throughout hospitalization. This may have prevented the progression of liver injury given the persistent toxic serum levels of APAP for approximately 60 hours.

To our best knowledge, this is the first case report that presents persistent toxic levels of APAP above the treatment line on the Rumack-Matthew curve nomogram, in a clinically asymptomatic patient with normal LFTs. In 2012, Dougherty and Klein-Schwartz reported a study of 20 patients with APAP levels that were initially below the treatment line on the nomogram but subsequently increased to toxic levels requiring antidote. Some patients were clinically symptomatic and displayed hepatotoxicity, and there was one event of a fatality. This study suggested that the nomogram may be limited in predicting toxicity based on a single plasma APAP concentration, especially in the context of overdosing on APAP-combination products. This is because opioid coingestion may delay gastric motility, yielding a second, higher plasma APAP concentration that warrants treatment based on the nomogram.^8^ Our case is unique in that the patient presented with an initial APAP serum level that required NAC treatment based on the Rumack-Matthew nomogram and received it despite no signs of hepatotoxicity throughout hospitalization.

In one prospective crossover study (Halcomb et al^9^), participants were divided into different groups based on whether they ingested 5 g of APAP, 5 g of APAP + 250 mg of diphenhydramine, or 5 g of APAP + 0.5 mg/kg of oxycodone. Subsequently, APAP concentrations were measured, and pharmacokinetic parameters were compared. First, the APAP-only ingesting group had a maximum APAP concentration of 71.8 μg/mL and the time to peak (tmax) was on average 1.71 hours. Second, the APAP and diphenhydramine coingested group reached a maximum APAP concentration of 67.9 μg/mL and the average tmax was 1.90 hours. Finally, the APAP and oxycodone coingested group reached a maximum APAP concentration of 42.9 μg/mL and tmax was 2.87 hours. Therefore, the group coingesting APAP and oxycodone had a 40% lower maximum APAP concentration and a 68% longer tmax, suggesting that oxycodone may delay the absorption of APAP and alter its pharmacokinetics.^10^

An alternative prospective observational study^10^ proposed opposing data to that by Halcomb et al.^9^ It postulated that serum APAP levels are crucial in determining the need for treatment with NAC and that although there are limited data available, opioid coingestion may not necessarily alter APAP pharmacokinetics. Participants were divided into 2 groups; one group consisted of APAP and opioid coingestion while the other ingested only APAP. The starting dose of APAP was 10 g for each. After 4.5 hours, both groups had similar APAP serum concentrations (56 vs 60 mg/L). The study suggested that serum APAP levels are not altered by opioid coingestion and that the true effect of opioids on APAP pharmacokinetics remains unclear.

Our case report follows a similar trajectory of another case study that presented a 65-year-old man with a history of bipolar disorder and suicide attempts. The patient was presented to the emergency department with altered mental status after ingesting oxycodone 5 mg/APAP 325 mg. The patient was admitted and managed in the intensive care unit with NAC intravenous therapy. Similar to our case, the patient's mental status improved and his LFTs remained normal throughout his entire hospitalization despite having 2 peaks in his serum APAP concentration levels. However, our case remains unique because our patient had toxic/peak APAP levels at 0, 12, 24, 36, and 48 hours after ingestion rather than a bimodal peak. The APAP levels became undetectable with 5 doses of NAC as recommended by the Poison Control Center. Both cases demonstrate the effect of concomitant oxycodone on delayed APAP absorption and, therefore, normal liver function tests with the administration of NAC in a timely and continuous manner.^7^

Studies on APAP toxicity in the setting of oxycodone or other combined products are limited. However, we present a case in which oxycodone-APAP induced an APAP overdose in a patient whose LFTs remained normal throughout hospitalizations. In addition, previous literature suggests that the Rumack-Matthew curve may be helpful for APAP toxicity alone, but may be less useful for predicting the course of toxicity in combination agents.^11^ However, we used the nomogram, and our patient had an initial plasma APAP concentration that was above the treatment line and, therefore, was treated with NAC promptly. This may have potentially saved the patient from experiencing acute liver injury or failure, but further literature and research studies are needed to explore this. In situations of an APAP overdose with a concomitant opioid, it may be helpful to continue NAC treatment until APAP levels become undetectable. However, more studies and reports of similar cases to medical literature are needed to further support these findings.

## DISCLOSURES

Author contributions: MA Haider and YS Gheit wrote the manuscript and did literature search. T. Nagi, C. Vallejo, ZK Suarez, OL Hernandez, and P. Gaisinskaya helped in data collection and manuscript elaboration. N. Markwart is supervising attending and did interpretation and critical revision of the manuscript for important intellectual content. MA Haider is the article guarantor.

Financial disclosure: None to report.

Informed consent was obtained for this case report.
